# An ensemble model for predicting dyslipidemia using 3-years continuous physical examination data

**DOI:** 10.3389/fphys.2024.1464744

**Published:** 2024-10-24

**Authors:** Naiwen Zhang, Xiaolong Guo, Xiaxia Yu, Zhen Tan, Feiyue Cai, Ping Dai, Jing Guo, Guo Dan

**Affiliations:** ^1^ School of Biomedical Engineering, Shenzhen University Medical School, Shenzhen University, Shenzhen, China; ^2^ Health Management Center, Shenzhen University General Hospital, Shenzhen University Clinical Medical Academy, Shenzhen University, Shenzhen, China; ^3^ Shenzhen Nanshan District General Practice Alliance, Shenzhen, China; ^4^ Department of Endocrinology and Metabolism, Shenzhen University General Hospital, Shenzhen, China

**Keywords:** dyslipidemia, prediction, physical examination data, machine learning, ensemble model

## Abstract

**Background:**

Dyslipidemia has emerged as a significant clinical risk, with its associated complications, including atherosclerosis and ischemic cerebrovascular disease, presenting a grave threat to human well-being. Hence, it holds paramount importance to precisely predict the onset of dyslipidemia. This study aims to use ensemble technology to establish a machine learning model for the prediction of dyslipidemia.

**Methods:**

This study included three consecutive years of physical examination data of 2,479 participants, and used the physical examination data of the first two years to predict whether the participants would develop dyslipidemia in the third year. Feature selection was conducted through statistical methods and the analysis of mutual information between features. Five machine learning models, including support vector machine (SVM), logistic regression (LR), random forest (RF), K nearest neighbor (KNN) and extreme gradient boosting (XGBoost), were utilized as base learners to construct the ensemble model. Area under the receiver operating characteristic curve (AUC), calibration curves, and decision curve analysis (DCA) were used to evaluate the model.

**Results:**

Experimental results show that the ensemble model achieves superior performance across several metrics, achieving an AUC of 0.88 ± 0.01 (*P* < 0.001), surpassing the base learners by margins of 0.04 to 0.20. Calibration curves and DCA exhibited good predictive performance as well. Furthermore, this study explores the minimal necessary feature set for accurate prediction, finding that just the top 12 features were required for dependable outcomes. Among them, HbA1c and CEA are key indicators for model construction.

**Conclusions:**

Our results suggest that the proposed ensemble model has good predictive performance and has the potential to become an effective tool for personal health management.

## 1 Introduction

Dyslipidemia has been recognized as a major risk factor for cardiovascular disease, which seriously endangers people’s health (Hedayatnia et al., 2020; Zhao et al., 2022; Doi et al., 2022). Over the past 3 decades, the global incidence of dyslipidemia has risen markedly, representing a grave threat to public health (Pirillo et al., 2021). A report from the World Health Organization found that 4.5% of the global mortality rate for people aged 18 and over and 2% of disability-adjusted life years are due to high cholesterol (Organization, 2021). Research suggests that the incidence density of dyslipidemia in China is as high as 101/1,000, 121/1,000 in men and 69/1,000 in women (Zhang et al., 2019). Cardiovascular events caused by high cholesterol in China have increased dramatically, and may reach 9.2 million between 2010 and 2030 (Moran et al., 2010). Dyslipidemia is defined as elevated plasma concentrations of total cholesterol (TC), low-density-lipoprotein-cholesterol (LDL-C), or triglycerides (TG), or a low plasma concentration of high-density-lipoprotein-cholesterol (HDL-C) or a combination of these features (Klop et al., 2013; Raja et al., 2023). Its complex pathogenesis, coupled with the absence of conspicuous symptoms in early stages, complicates its detection and often leads to its underestimation. Therefore, the prediction of dyslipidemia occurrence plays an important role in improving its preventive and therapeutic effects.

In recent years, several studies have investigated the primary risk factors associated with dyslipidemia, including body mass index (BMI), waist-to-hip ratio, obesity, and gender, yielding significant insights (Vekic et al., 2019; Kavey, 2023; Ruan et al., 2024). Qi et al. (Qi et al., 2015) analyzed 5,375 participants aged 18 and older to ascertain the prevalence of dyslipidemia and its associated risk factors. Similarly, Ni et al. (Ni et al., 2015) employed a multi-stage stratified cluster random sampling approach to survey 1,995 adults, averaging 46.56 years in age. The findings indicate a substantial correlation between dyslipidemia and factors such as age, smoking, hypertension, diabetes, and BMI. Although these studies have helped identify risk factors for dyslipidemia, they do not have the ability to predict the long-term risk of dyslipidemia. Some studies have noted the limitations of these methods and proposed various approaches for prediction using logistic regression or Cox proportional hazards models (Kavey, 2023; Lai et al., 2022; Wang J.-S. et al., 2022; Kim et al., 2021; Lan et al., 2023). As comprehension of health outcomes’ complexity deepens, it becomes evident that traditional models, limited by their inability to account for non-linear associations, fall short of accurately encapsulating health outcome intricacies (De Silva et al., 2020).

Machine learning (ML) is a powerful computer-assisted data mining and analysis method that can handle large, complex, and diverse data. It has been widely used in healthcare applications, including disease risk prediction and medical diagnosis (Li et al., 2023; Ibrahim and Abdulazeez, 2021). ML has powerful nonlinear fitting capabilities and can solve this problem well. Previous studies (Zhang et al., 2019; Sasagawa et al., 2024) have developed some prediction models for dyslipidemia using algorithms such as the random survival forest model, demonstrating the ML’s potential in predicting dyslipidemia. Despite these advancements, the application of ML methods in dyslipidemia prediction remains underexplored. These studies also have some shortcomings, such as the reliance on a singular prediction model and the lack of comprehensive validation of different models, making it hard to ensure the stability and applicability of the methodology. Ensemble technology uses the excellent integration ability of the meta-learner on the results of the base learners to achieve more effective performance than a single model. It has been successfully applied in prediction tasks (Lu et al., 2024; Sun et al., 2024; Zhang et al., 2022).

Therefore, in this study, we aimed to use ensemble technology to develop a reliable dyslipidemia prediction model. By integrating the advantages of different machine learning models and making full use of 3 years of continuous physical examination data of non-diseased people, an ensemble model that can effectively predict dyslipidemia was constructed.

## 2 Materials and methods

### 2.1 Participants and data collection

**FIGURE 1:**
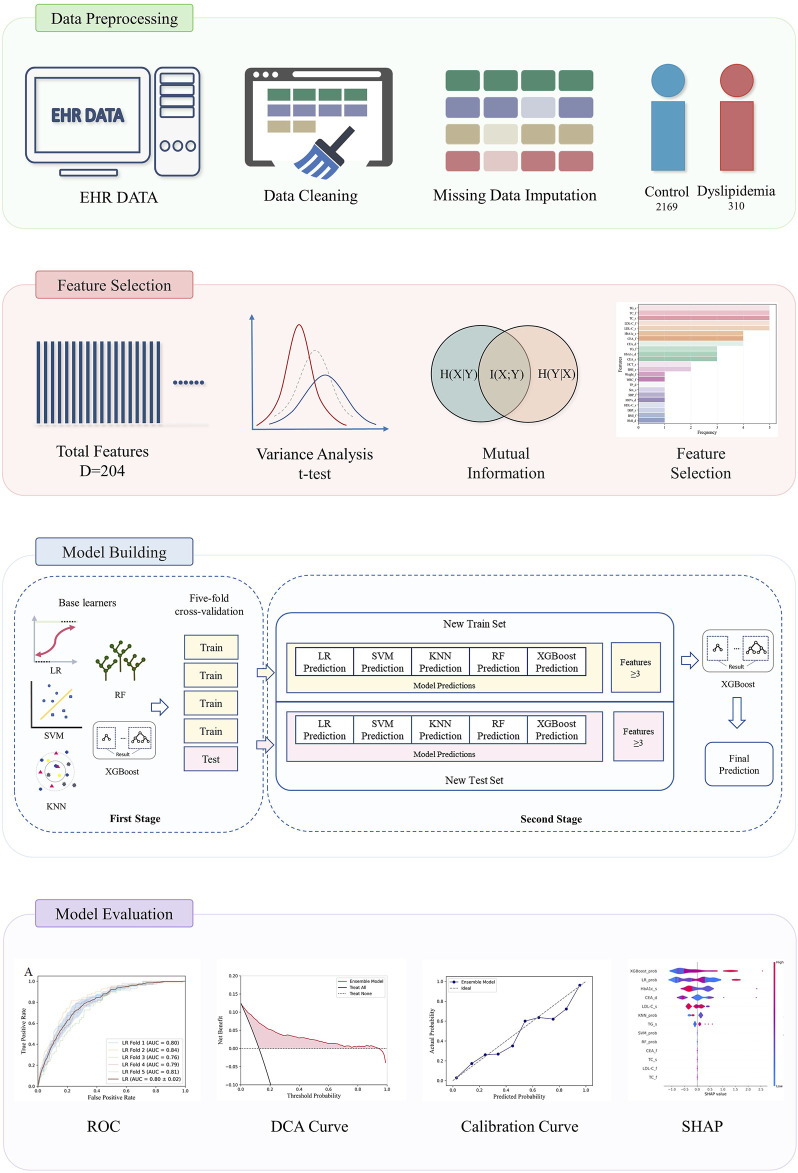
The analysis workflow for prediction of dyslipidemia from EHR data.

The overall process of the experiment is shown in Figure 1. We used medical examination data provided by Shenzhen University General Hospital, China, covering the period from December 2018 to December 2022. All participants received a medical examination at the hospital. Ethical approval was obtained from the Ethics Committee of Shenzhen University, Shenzhen (approval number: PN-202300093). Informed consent was waived due to the retrospective nature of the study. The research adhered to the principles outlined in the Declaration of Helsinki.

The electronic medical records of participants with a history of undergoing multiple years’ worth of physical examinations were meticulously reviewed. Our inclusion criteria comprised two distinct categories of physical examination subjects, i.e., individuals exhibiting consistent normal blood lipid levels across three consecutive physical examinations, and those with normal blood lipid levels during the initial two physical examinations but displaying abnormal blood lipid levels in the subsequent third examination. By including these two different participants in the study, we aim to comprehensively understand the occurrence and development mechanism of dyslipidemia, and provide a more accurate reference for future intervention and prediction. In accordance with the 2023 China guidelines for lipid management (Jian-Jun et al., 2023), dyslipidemia was precisely defined as the presence of TC ≥ 5.2 mmol/L, TG ≥ 1.7 mmol/L, LDL-C ≥ 3.4 mmol/L, and/or HDL-C < 1.0 mmol/L.

The data content is primarily categorized into two groups: demographic data and laboratory test results. Every physical examination will meticulously document individual demographic information, encompassing age, gender, height, weight, physical examination date, blood pressure, pulse rate, and BMI. Laboratory findings are likewise derived from each physical examination record. The encompassing examination indicators comprise blood cell analysis, urinalysis, tumor markers, blood glucose test, blood lipid test, liver function, kidney function, and thyroid function.

### 2.2 Development and validation of machine learning models

#### 2.2.1 Machine learning models

In this study, five different machine learning models were employed for predictive modeling of dyslipidemia, namely, support vector machine (SVM), logistic regression (LR), random forest (RF), K nearest neighbor (KNN) and extreme gradient boosting (XGBoost).

SVM (Vapnik, 1999) is a powerful machine learning algorithm that can be used to solve classification problems. The core idea of SVM is to find an optimal decision boundary, which can divide different categories of datapoints in the feature space.

LR (Cox, 1958) is a statistical method commonly used to solve binary classification problems. Logistic regression models map the output values to a range between 0 and 1 by passing linear combinations of independent variables to logistic functions, which not only provide a prediction of the occurrence of an event, but also account for the effect of independent variables on the probability of an event.

RF (Breiman, 2001) is a powerful ensemble learning algorithm that is widely used in classification tasks. It works based on the construction of multiple decision trees, each based on a different subset of data and features, which helps to reduce the risk of overfitting and improve the robustness of the model.

KNN (Fix and Hodges, 1989) is a supervised learning algorithm that is widely used in classification problems by using information from neighbors to make predictions. It is based on the proximity between samples, especially using the labels of the K closest training samples to make predictions.

XGBoost (Chen and Guestrin, 2016) is a widely used ensemble learning method that performs well in a variety of machine learning tasks. The core principle of XGBoost is to iteratively add new weak models to correct the errors of the model in the previous round of iterations and build an efficient prediction model.

Each of the above machine learning models has been carefully configured to achieve accurate prediction of dyslipidemia. Specifically, we first selected the commonly used hyperparameters and candidate values that need to be optimized for each model, then used grid search to optimize the hyperparameters of each model, and used five-fold cross validation to select the best parameters. The specific parameter selection and optimization results are shown in Table 1. In addition, based on the above five machine learning models, ensemble technology will be used to effectively fuse their prediction results to achieve more accurate prediction performance.

**TABLE 1:** Specific parameter selection and optimization results of each model.

Model	Model parameter	Range	Parameter after optimization
SVM	C	[0.1, 0.5, 1]	0.1
	kernel	[“rbf”, “linear”, “poly”]	“linear”
	gamma	[0.05, 0.1, 0.15]	0.1
LR	C	[50, 100, 150]	100
	max_iter	[1,000, 2000, 3,000]	2,000
	solver	[“lbfgs”, “newton-cholesky”, “sag”]	“newton-cholesky”
RF	n_estimators	[10, 15, 20]	15
	max_depth	[6, 8, 10]	10
	max_features	[“sqrt”, “log2”]	“sqrt”
KNN	n_neighbors	[20, 30, 40]	40
XGBoost	n_estimators	[10, 50, 100]	50
	max_depth	[2, 4, 6]	2
	learning_rate	[0.05, 0.1, 0.15]	0.05

#### 2.2.2 Feature selection

In this study, we first addressed the heterogeneity in physical examination items across subjects by excluding those for which data were available for less than one-third of the cohort. For the remaining dataset, missing values were imputed using either mean or mode, depending on the nature of the data, thus completing the data preprocessing phase. We then defined the sets of index values for each subject in the first and second years as $X_{f}$ and $X_{s}$ , respectively, and used $X_{f}$ , $X_{s}$ , and their difference $D=X_{s}-X_{f}$ as features, identifying a total of 204 features. Recognizing the potential for irrelevant or redundant information within these features, the study implemented a two-step feature selection strategy. Firstly, we used t-test or $\chi^{2}$ test to identify features that showed significant differences between the dyslipidemia group and the non-dyslipidemia group, excluding those with *P*-values above 0.05. We then applied mutual information to remove redundant features, ensuring that the selected features were both statistically significant and independent across the groups. We explored the optimal number of features (N) to minimize redundancy while retaining sufficient discriminatory information. This approach enabled us to investigate the optimal number of features required to maintain model performance, experimenting with N values in increments of two from 2 to 20. This process facilitated effective feature selection, optimizing the efficiency of feature utilization.

#### 2.2.3 Model building and evaluation

To accurately predict dyslipidemia onset utilizing routine physical examination data, this study introduced a stacking ensemble model executed in two stages. The first stage employed five base learners, including LR, SVM, KNN, RF, and XGBoost, to produce preliminary outputs. These outputs, alongside selected key features, serve as inputs for the second stage. The second stage integrated these inputs to train and establish the final predictive model.

In the first stage, to mitigate the risk of overfitting, each base learner underwent training and evaluation employing a five-fold cross-validation approach. This entailed partitioning the data into five subsets, with each subset serving once as the test set while the model trains on the remaining four. The model then predicted outcomes for both the training and test sets, generating sets of predictions for each. Concurrently, key features were identified based on their recurrence, with those appearing more than three times across five folds deemed significant. The outputs from this stage, comprising both the predicted values and the identified key features for the training and test sets, were then forwarded as inputs to the second stage. Moreover, an analysis to assess the impact of varying the number of features on model performance was conducted, aiming to ascertain the most effective feature set for the predictive model.

In the second stage, based on the results of each base learner in the first stage, XGBoost was chosen to develop the ensemble model for final predictions. To ensure robustness and validity, the five-fold cross-validation technique was reapplied. The training dataset encompassed the predictive outcomes and pivotal features from the first stage, generated by the five base learners in the best feature set. Similarly, the test dataset was constituted of analogous predictions and features, also from the same base models. This construction of new training and test datasets addresses and circumvents potential issues of data leakage. Ultimately, the ensemble model, having been thoroughly trained, performs the final predictive analysis on the test dataset.

### 2.3 Statistical analysis

Clinical factors were analyzed using Student’s t-test, Mann-Whitney U test, or Chi-square test according to the data distribution. Multiple criteria, including sensitivity, specificity, accuracy, and the area under the ROC curve (AUC), were used to evaluate the effectiveness of these models. Calibration curve and decision curve analysis were used to evaluate clinical usability. In addition, to facilitate understanding of the contribution of the second stage model input features to the prediction score, we calculated the SHapley Additive exPlanations (SHAP) values and illustrated them graphically. Statistical analyses were performed using Python (version 3.9) or Medcalc (version 22).

## 3 Results

### 3.1 Participant characteristics

The dataset encompasses 7,437 distinct medical examination records from a total of 2,479 participants. Participants were categorized based on outcomes from their third examination into two sets: dyslipidemia, comprising 310 individuals or 12.5% of the study population, and non-dyslipidemia, numbering 2,169 or 87.5% of the total. The characteristics of the dyslipidemia and non-dyslipidemia sets were shown in Table 2. As shown, there were differences in the baseline data between dyslipidemia and non-dyslipidemia in some characteristics, indicating that the development of dyslipidemia was traceable. In addition to the physical examination data listed in Table 2, there were also blood cell analysis, urinalysis, blood glucose test, liver function, kidney function, and thyroid function. The baseline data of these characteristics were in Supplementary Table S1.

**TABLE 2:** Baseline characteristics of dyslipidemia and non-dyslipidemia participants.

Characteristics	Dyslipidemia (n = 310)	Non-dyslipidemia (n = 2,169)	*P*-value
Age	32.00 (28.00, 37.00)	33.00 (29.00, 38.00)	0.106
Sex			<0.001
Male	159 (51.29%)	856 (39.47%)	
Female	151 (48.71%)	1,313 (60.53%)	
Height	164.00 (159.00, 170.50)	166.00 (159.00, 172.00)	0.021
Weight	57.40 (51.70, 65.80)	60.90 (53.30, 70.30)	<0.001
SBP	113.00 (105.00, 122.00)	116.00 (108.00, 127.00)	<0.001
DBP	68.00 (63.00, 75.00)	70.00 (65.00, 77.00)	<0.001
Pulse	79.00 (72.00, 88.00)	79.00 (72.00, 88.00)	0.754
BMI	21.40 (19.70, 23.40)	21.97 (20.20, 24.50)	<0.001
TC	4.03 (3.70, 4.34)	4.45 (4.17, 4.73)	<0.001
TG	0.79 (0.63, 1.01)	0.99 (0.75, 1.23)	<0.001
HDL-C	1.54 (1.34, 1.75)	1.45 (1.22, 1.78)	0.002
LDL-C	2.50 (2.17, 2.84)	2.92 (2.63, 3.17)	<0.001
HbA1c	5.30 (5.10, 5.40)	5.30 (5.10, 5.50)	0.314
CEA	1.39 (0.95, 1.99)	1.56 (1.16, 2.19)	0.001

*P* < 0.050 is considered statistical significance. SBP, systolic blood pressure; DBP, diastolic blood pressure; TC, total cholesterol; TG, triglycerides; HDL-C, high-density lipoprotein cholesterol; LDL-C, low-density lipoprotein cholesterol; HbA1c, glycated hemoglobin; CEA, carcinoembryonic antigen. Categorical variables, expressed as frequencies (proportions), line χ2 test. Non-normally distributed variables, expressed as median (interquartile range), line Mann–Whitney U test.

### 3.2 Feature selection

**FIGURE 2:**
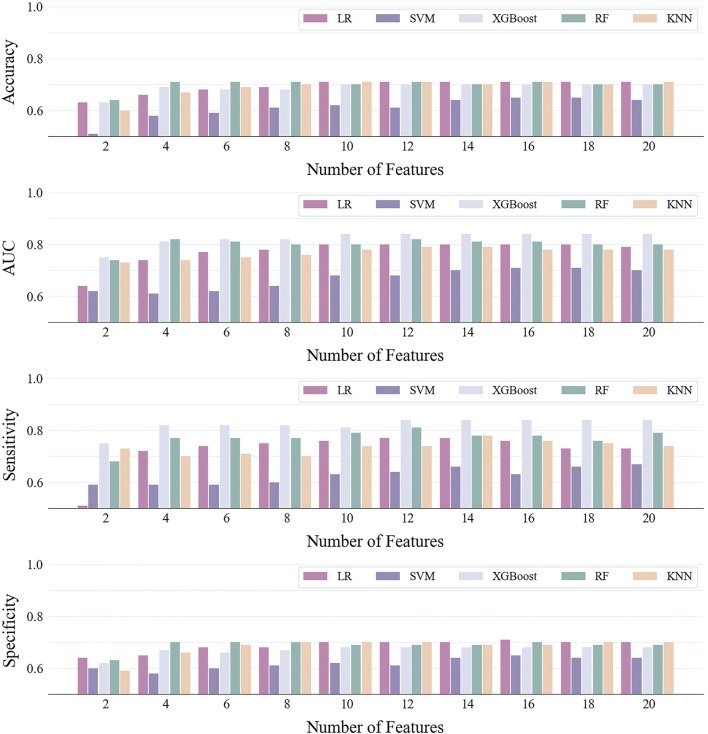
Predictive performance of different base learners and different number of features.

In the first stage, the construction of models involved the application of various base learners alongside different numbers of feature. The results were shown in Figure 2 and the quantitative description of the results was in Supplementary Table S2. In all base learners, the prediction performance improves with the increase in features and then reaches a plateau. When the number of features was 12, the models generally achieved the best performance. Notably, disparities in performance were observed among the base learners, even with identical feature sets. In particular, the XGBoost model (AUC = 0.84, number of features was 12) demonstrated superior predictive accuracy compared to the SVM model (AUC = 0.68, number of features was 12), which lagged in performance. This result showed that selecting appropriate models and features can effectively improve the accuracy of dyslipidemia prediction.

### 3.3 Feature importance

**FIGURE 3:**
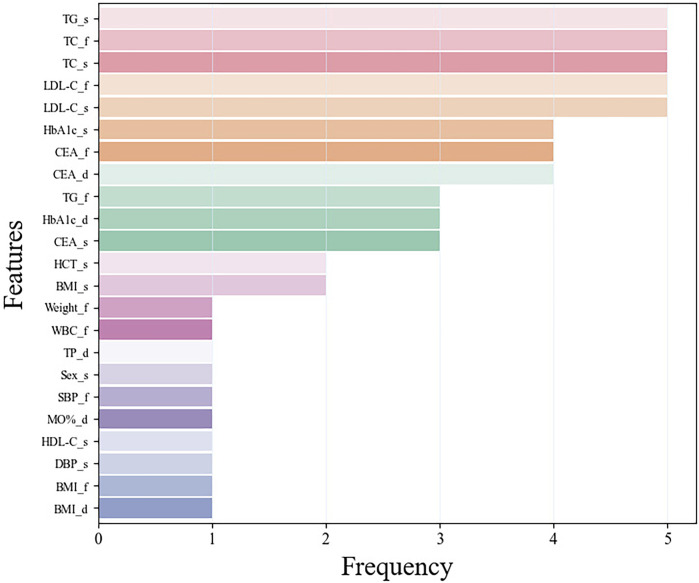
The frequency of features using in the five-fold cross-validation. Among them, the suffix f means the first examination, s means the second examination, and d means the difference between the two examinations.

Following the performance evaluation of base learners, we also conducted a deep investigation on feature utilization, specifically focusing on scenarios where the feature number was set to 12. We tallied the frequency with which each feature was selected across the five-fold cross-validation process. This examination’s findings were illustrated in Figure 3. The analysis unveiled a notable consistency in feature usage across the various folds: five features (TC and LDL-C at the first examination, TG, TC and LDL-C at the second examination) were employed in all five folds of validation, while three features (Glycated hemoglobin (HbA1c) at the second examination, carcinoembryonic antigen (CEA) at the first examination, and the difference between the two CEA examinations) were utilized in four out of five validations. Additionally, we analyzed the top 12 features in each fold during the five-fold cross-validation. Supplementary Figure S3 presents the mutual information scores for these 12 features during feature selection. The features with higher mutual information scores are also those mentioned above. This result showed that the model’s robust consistency and stability throughout different segments of validation. Among the frequently utilized indicators, TC, LDL-C, TG, CEA, and HbA1c were distinguished as key features, reflecting their significant role in the model’s predictive capability.

### 3.4 Development and validation of prediction models

**FIGURE 4:**
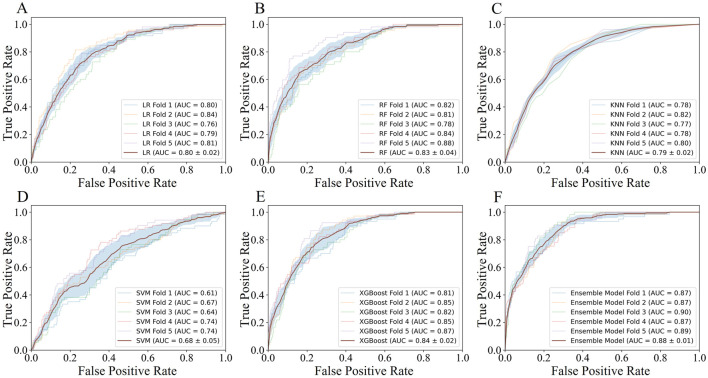
The AUC performance and average AUC performance of base learners and ensemble model in five-fold cross validation.

Employing the predicted outcomes from the base learners alongside the key features, the inputs for the ensemble model were synthesized, culminating in the final predictive analysis conducted using the XGBoost algorithm. Using ROC curve analysis, we calculated the corresponding AUCs for the different base learners and ensemble model when the number of features was 12 in five-fold cross validation. As can be seen in Figure 4, the AUC scores for the base learners fluctuate between 0.68 ± 0.05 and 0.84 ± 0.02, whereas the ensemble model achieved an AUC of 0.88 ± 0.01 (*P* < 0.001), markedly surpassing those of the individual base learners. Table 3 showed a more detailed average performance comparison. The ensemble model exhibited pronounced superiority in several performance metrics, with accuracy of 0.78 ± 0.01 and specificity of 0.78 ± 0.02, both of which were better than other base learners. Additionally, we conducted experiments by adjusting the ratio of dyslipidemia and non-dyslipidemia samples under the same hyperparameters, and the results showed that the model maintained good predictive performance across different sample ratios (Supplementary Table S3). These insights not only highlighted the capability of machine learning techniques in dyslipidemia predictions but also illustrated the profound impact of ensemble learning approach on improving predictive accuracy.

**TABLE 3:** Average prediction performance of different machine learning models in five-fold cross validation.

Models	Accuracy (mean ± SD)	AUC (mean ± SD)	Sensitivity (mean ± SD)	Specificity (mean ± SD)	*P*-value
LR	0.71 ± 0.01	0.80 ± 0.02	0.77 ± 0.07	0.70 ± 0.01	<0.001
RF	0.72 ± 0.02	0.83 ± 0.04	0.77 ± 0.05	0.71 ± 0.02	<0.001
KNN	0.71 ± 0.01	0.79 ± 0.02	0.74 ± 0.08	0.70 ± 0.02	<0.001
SVM	0.61 ± 0.10	0.68 ± 0.05	0.64 ± 0.12	0.61 ± 0.13	<0.001
XGBoost	0.70 ± 0.03	0.84 ± 0.02	**0.84 ± 0.07**	0.68 ± 0.03	<0.001
Ensemble Model	**0.78 ± 0.01**	**0.88 ± 0.01**	0.80 ± 0.06	**0.78 ± 0.02**	<0.001

Values in bold indicate best performance

### 3.5 Clinical usage of the models

**FIGURE 5:**
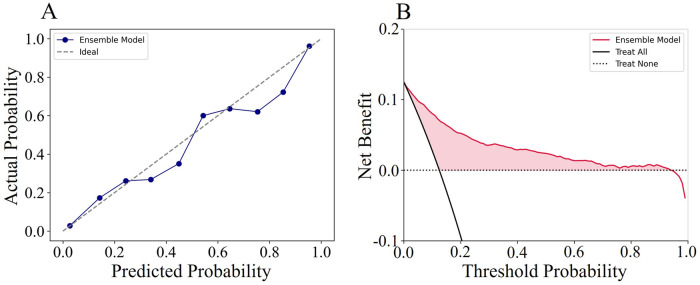
The calibration curves and decision curve analysis curves of the ensemble model.

To visually demonstrate the clinical usability of the ensemble model, we plotted calibration curves and conducted decision curve analysis (DCA). The calibration curve showed that the actual observations were well consistent with the predictions of the ensemble model (Figure 5A), suggesting that the ensemble model has an excellent predictive value. The DCA curve of the ensemble model also demonstrated good clinical utility, showing preferable positive net benefit (Figure 5B). In addition, similar results were shown in each fold of the five-fold cross validation (Supplementary Figures S1, S2).

### 3.6 Model explainability

**FIGURE 6:**
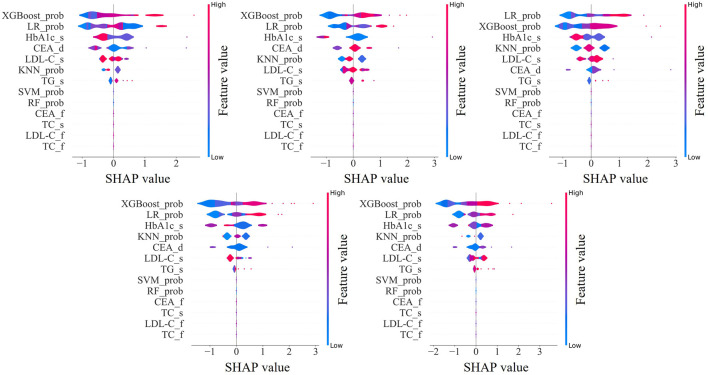
SHapley Additive exPlanations summary plot of the input features in second stage model. XGBoost_prob, LR_prob, KNN_prob, SVM_prob, and RF_prob are the probabilities corresponding to the first stage XGBoost, LR, KNN, SVM, and RF models; HbA1c, glycated hemoglobin; CEA, carcinoembryonic antigen; LDL-C, low-density-lipoprotein-cholesterol; TG, triglycerides; TC, total cholesterol. The suffix f means the first examination, s means the second examination, and d means the difference between the two examinations.

We visualized the influence of predictor variables on the results based on SHAP plots. Figure 6 shows the SHAP summary plot of the second stage model input features in five-fold cross-validation. Specifically, the influence of variables on the results can be intuitively explained by the magnitude of the SHAP value (indicated by color change) and the trend on the horizontal axis of the variable (the probability of an adverse outcome). For example, in the scenario of HbA1c_s, individuals with higher indicators (indicated in red) were more likely to have dyslipidemia (on the right) compared to those with lower HbA1c_s indicators (indicated in blue). Overall, it is evident that the important predictors of these five models have strong consistency, among which XGBoost_prob, LR_prob, HbA1c_s, CEA_d, LDL-C_s, KNN_prob, and TG_s were extracted as important predictors.

## 4 Discussion

Dyslipidemia has become a common disease among patients, posing a significant risk for the development and progression of cardiovascular disease and is one of the most important risk factors for atherosclerotic cardiovascular disease, which accounts for the most deaths worldwide (Sandesara et al., 2019). Therefore, early risk prediction is particularly important for the prevention and management of dyslipidemia. In this research, we developed an ensemble model tailored to predict dyslipidemia risk in the third year based on data from the initial 2 years’ physical examinations. The efficacy of this model was corroborated on a dataset encompassing 2,479 participants, where it attained an AUC value of 0.88 ± 0.01 (*P* < 0.001), indicating a high capacity for dyslipidemia prediction. The improved performances of the ensemble model over the base learners were consistent with our assumption that ensemble model performs better than individual machine learning models. Different models are suited to handling different types of data patterns. For instance, LR is well-suited for linear relationships, RF and XGBoost excel at handling nonlinear data, SVM performs well with high-dimensional data, and KNN are effective at capturing local patterns. By combining these algorithms (LR, SVM, RF, KNN, and XGBoost) into an ensemble model, we can leverage the strengths of each algorithm and compensate for their individual weaknesses, leading to significantly improved predictive performance.

Furthermore, the investigation delved into identifying the optimal minimal set of features necessary for accurate predictions. Through rigorous application of statistical analyses and mutual information for feature selection, the study identified that a subset of the top 12 features suffices to achieve reliable predictive outcomes. In the statistical examination of the 12 features utilized in the modeling process by base learners, it was observed that five features were consistently selected across the five-fold cross-validation, whereas an additional three features were chosen in four out of five folds. These eight key features encompass TC, LDL-C, and CEA from the first physical examination; TC, TG, LDL-C, and HbA1c from the second examination; along with the difference in CEA levels between the two examinations.

Notably, TC, TG, and LDL-C were acknowledged as fundamental metrics for assessing blood lipid status, with HbA1c also recognized for its association with lipid concentrations (Li et al., 2022; Bulut et al., 2017). Previous study (Feng et al., 2019) have shown that high LDL-C is the most common component of dyslipidemia, followed by elevated TG. HbA1c was a recognized indicator related to dyslipidemia, and it was significantly correlated with common lipid parameters such as TC, TG, and LDL-C (Ozder, 2014; Reddy et al., 2014). Previous study (Huang et al., 2021) have shown that lowering HbA1c levels may improve blood lipid levels. At the same time, HbA1c was closely related to diabetes, and abnormal lipid metabolism was part of the pathogenesis of diabetes (Sunjaya and Sunjaya, 2018). Metabolic syndrome was a combination of metabolic abnormalities such as hypertension, obesity, hyperglycemia, and dyslipidemia, which increases the risk of cancer (Mendrick et al., 2018). CEA was widely considered to be a serological tumor marker, and CEA levels can affect a variety of metabolic diseases (Lu et al., 2018; Wang C.-H. et al., 2022). Therefore, CEA levels may have a certain relationship with dyslipidemia, which was consistent with the results of the model. The inclusion of these indicators as key features demonstrates the model’s strong clinical relevance and interpretability.

Moreover, the importance of predictors in the ensemble model evaluated using SHAP values was consistent across five-fold cross validation. Among them, the prediction probabilities of the XGBoost, LR, and KNN models in the first stage were important predictors of ensemble models, which proved that the ensemble model can well integrate the advantages of each base learner and achieve better prediction performance. In addition, an interesting phenomenon was observed that HbA1c and CEA were more important than TC, TG, and LDL-C, which were conventionally considered predictors of dyslipidemia. This may be because the ensemble model does not obtain results based on a simple linear relationship, but explores a more complex relationship between predictors and results.

Current research into dyslipidemia predominantly centers on elucidating its risk factors. For example, Qi et al. (Qi et al., 2015) and Ni et al. (Ni et al., 2015) undertook analyses using different datasets and statistical methodologies to investigate dyslipidemia prevalence and the differential indicators between affected individuals and the general populace, with the objective of identifying dyslipidemia risk factors. However, such cross-sectional investigations are largely constrained to singular temporal analyses, neglecting the longitudinal progression of dyslipidemia, which curtails their predictive utility. Conversely, our research examines the dynamic evolutions of physiological indicators over time. Through a longitudinal analysis of indicator fluctuations within the same cohort across multiple intervals, we discern patterns indicative of alterations in lipid concentrations, thereby facilitating effective dyslipidemia prediction. While several studies have employed cohort data for dyslipidemia predicting (Sasagawa et al., 2024), the majority are limited by their reliance on singular predictive model, overlooking the varied data mining emphases inherent to different algorithms. Their performance, as measured by the AUC, usually hovers around 0.83. Our methodology diverges by adopting a multifaceted perspective, substantially augmenting predictive efficacy through the exploitation of diverse model strengths and their integration. This strategy not only elevates the accuracy of our predictive model but also its applicability in real-world settings, furnishing a robust scientific foundation for dyslipidemia’s early prevention and management.

The primary application of this model is in health examination centers, where it can be used to predict the risk of dyslipidemia in the following year based on consecutive years of health examination data. Examination centers need to maintain continuous health records for each patient, and by analyzing both historical and current examination data, the model can provide predictions on the likelihood of developing dyslipidemia in the future. This model not only provides early warnings of dyslipidemia for patients, but also reinforces the value of regular health check-ups, thereby encouraging patients to adhere to scheduled examinations. For health examination centers, the model offers more personalized services, enhancing customer satisfaction. Moreover, this modeling approach can be extended to risk prediction for other diseases, showcasing its broad clinical application potential.

The strength of this study lies in the integration of multiple machine learning algorithms to construct an ensemble model for dyslipidemia prediction. In comparison to base learners, including LR, SVM, RF, KNN, and XGBoost, our ensemble model has shown an improvement in the AUC indicator, with the AUC improved by 0.04–0.20. In addition, we also conducted a detailed analysis of the features selected by the model to ensure transparency and facilitate the interpretation of the results. This study provides a health management tool that can help identify individuals at risk of dyslipidemia early, potentially reducing its prevalence. However, this study has several limitations. Firstly, the data samples were exclusively sourced from Shenzhen City, Guangdong Province, China, which may impart a regional bias to the findings. It is worth noting that Shenzhen is a city with a large migrant population, resulting in a relatively diverse demographic composition. Therefore, the impact of regional and demographic characteristics on the results may not be as significant as in other areas. Secondly, the median age of the participants is 32 years, predominantly under 50, leading to an underrepresentation of the elderly demographic in the analysis. These limitations underscore the necessity for subsequent studies to encompass a more diverse and representative population sample and to explore alternative methods of feature construction. Such expansions are crucial for augmenting the model’s generalizability and enhancing its predictive precision.

## 5 Conclusion

In conclusion, we presented an ensemble learning approach to predict dyslipidemia risk in the third year based on physical examination data from two successive years. The empirical findings substantiate the effectiveness of our proposed methodology in accurately predicting dyslipidemia, with the model also exhibiting notable clinical interpretability. This study also found that HbA1c and CEA could be used as key indicators for assessing blood lipid status. Future directions include refining the model through the inclusion of a more extensive population sample and investigating the potential for more efficient exploitation of existing features or the innovation of new feature engineering strategies to elevate the predictive accuracy for dyslipidemia.

## Data Availability

The raw data supporting the conclusions of this article will be made available by the authors, without undue reservation.
