# Supplementary material for: An ensemble model for predicting dyslipidemia using 3-years continuous physical examination data
Source: Front Physiol. 2024 Oct 24;15:1464744. doi: 10.3389/fphys.2024.1464744 (PMC11540663; doi:10.3389/fphys.2024.1464744)
Supplement: Supplementary file 1 [file Table1.DOCX]

Supplementary Material

# Supplementary Figures and Tables

## Supplementary Figures


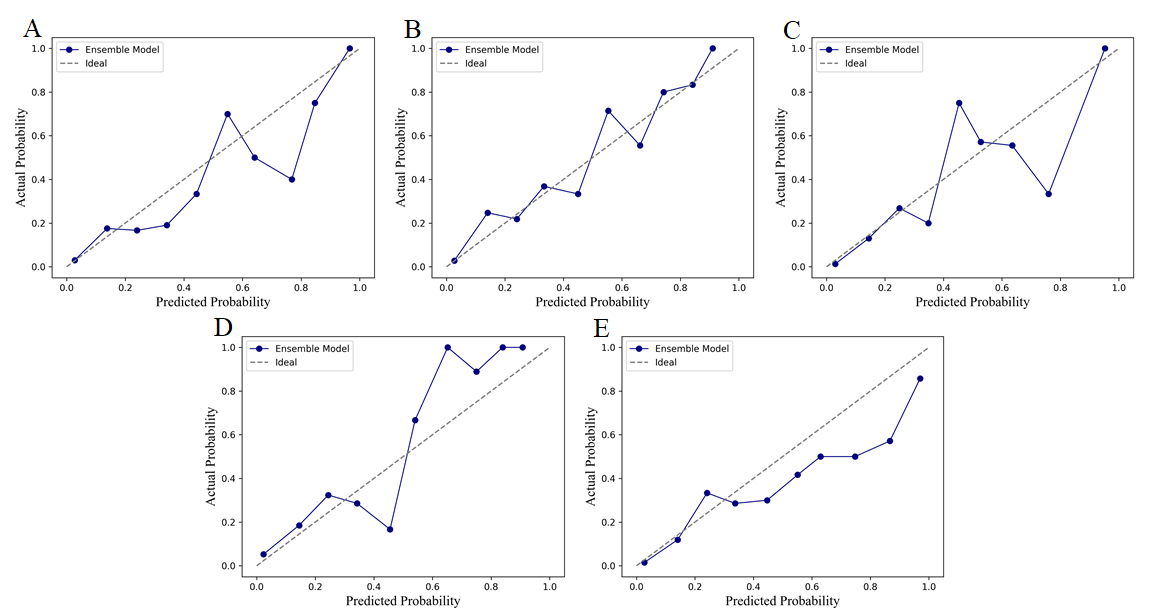


**Supplementary Figure 1.** The calibration curves of the ensemble model in five-fold cross validation.


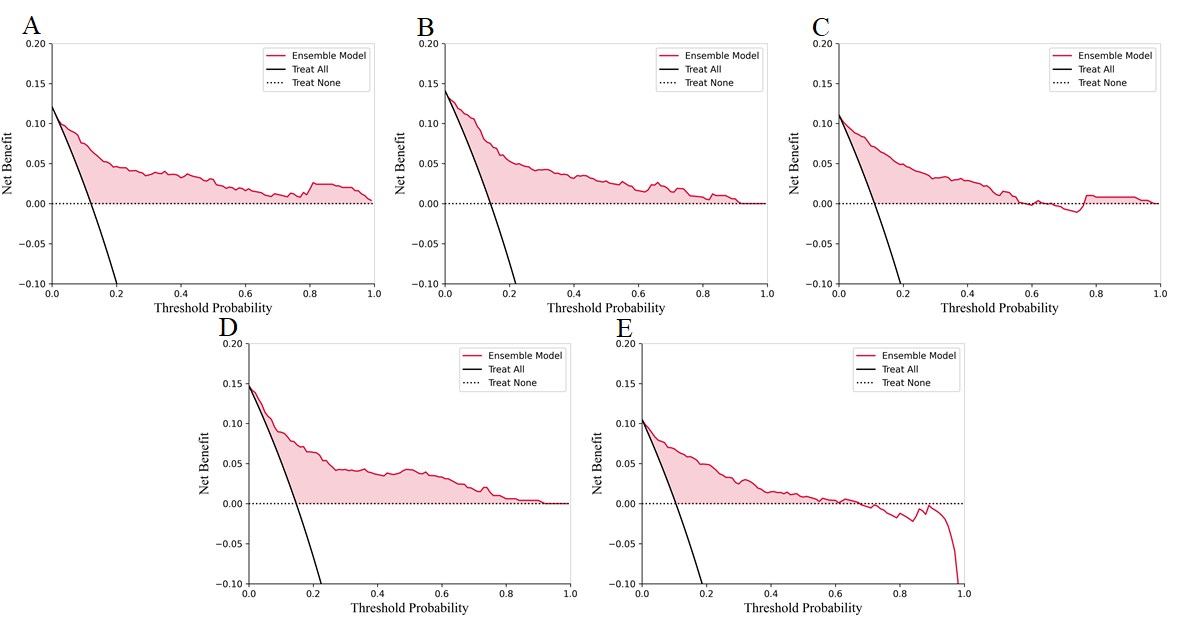


**Supplementary Figure 2.** The decision curve analysis curves of the ensemble model in five-fold cross validation.


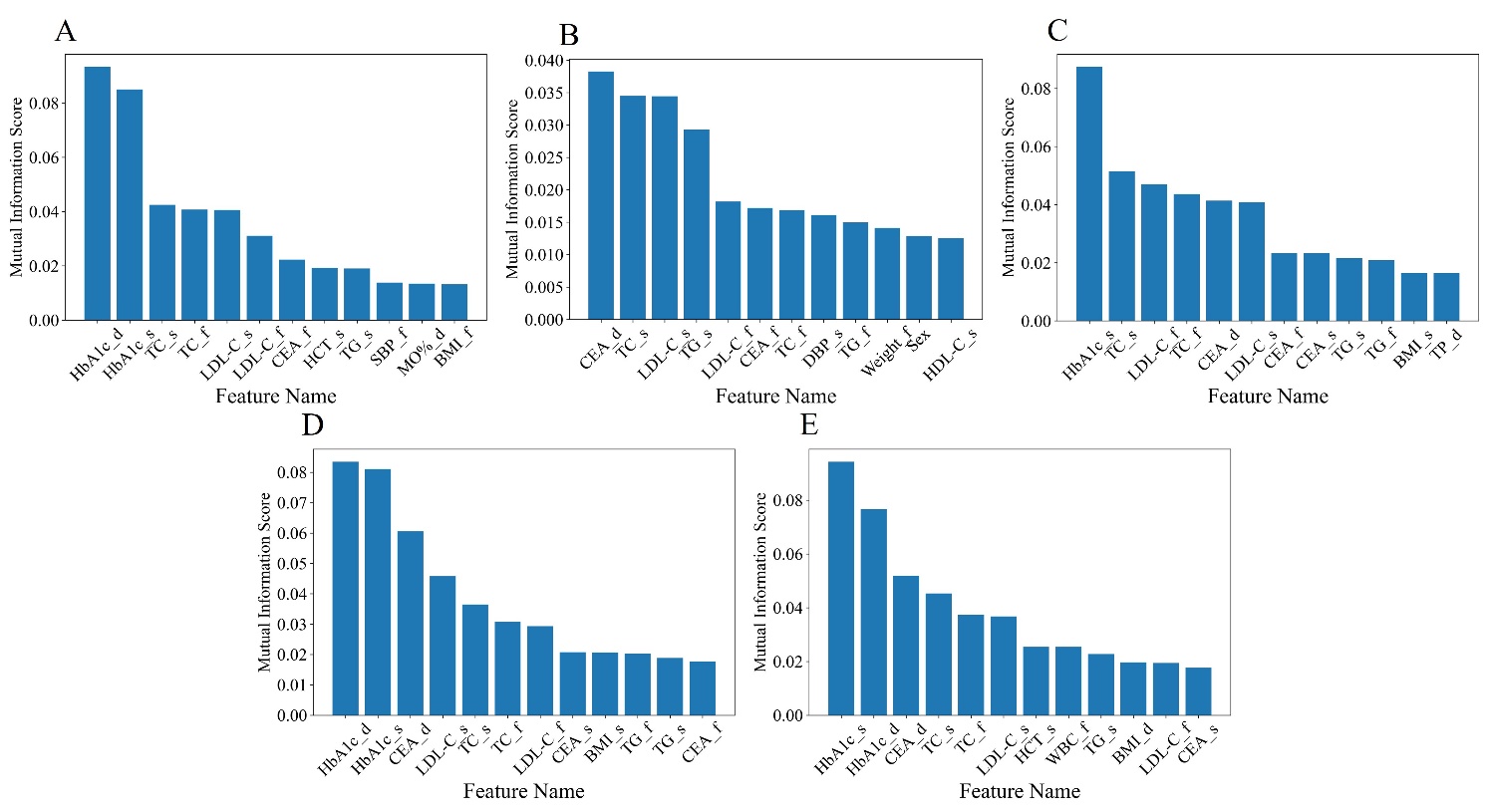


**Supplementary Figure 3.** The mutual information scores for top 12 features during feature selection in five-fold cross validation.

## Supplementary Tables

**Supplementary Table 1** Baseline characteristics of dyslipidemia and non-dyslipidemia participants

| Characteristics | Dyslipidemia (n=310) | Non-Dyslipidemia (n=2169) | *P*-value |
| --- | --- | --- | --- |
| total protein | 76.60 (74.00, 79.30) | 77.14 (73.19, 81.08) | 0.036 |
| albumin | 49.00 (47.30, 50.80) | 49.38 (46.62, 52.14) | 0.110 |
| globulin | 27.50 (25.30, 29.80) | 27.76 (24.52, 31.00) | 0.423 |
| gamma-glutamyl transpeptidase | 14.00 (11.00, 20.00) | 17.00 (13.00, 25.00) | ＜0.001 |
| alkaline phosphatase | 59.00 (50.00, 71.00) | 61.00 (52.00, 72.00) | 0.203 |
| total bilirubin | 11.50 (8.60, 15.50) | 11.40 (8.53, 15.60) | 0.970 |
| direct bilirubin | 4.70 (3.70, 6.00) | 4.55 (3.70, 5.90) | 0.213 |
| indirect bilirubin | 6.70 (4.80, 9.40) | 6.75 (4.93, 9.98) | 0.495 |
| alanine aminotransferase | 14.00 (10.00, 20.00) | 15.00 (11.00, 23.00) | 0.001 |
| aspartate aminotransferase | 16.00 (14.00, 20.00) | 17.50 (15.00, 21.00) | 0.001 |
| albumin/globulin ratio | 1.80 (1.60, 2.00) | 1.80 (1.60, 2.00) | 0.971 |
| aspartate aminotransferase/ alanine aminotransferase | 1.20 (0.90, 1.50) | 1.10 (0.90, 1.40) | 0.005 |
| absolute value of lymphocytes | 1.94 (1.64, 2.28) | 1.98 (1.70, 2.31) | 0.100 |
| absolute neutrophil count | 3.18 (2.58, 3.87) | 3.50 (2.74, 4.11) | 0.001 |
| platelet specific volume | 0.26 (0.23, 0.30) | 0.26 (0.23, 0.30) | 0.271 |
| monocyte ratio | 6.50 (5.50, 7.60) | 6.40 (5.60, 7.60) | 0.704 |
| eosinophil ratio | 1.80 (1.10, 2.90) | 1.80 (1.10, 3.08) | 0.806 |
| basophil ratio | 0.60 (0.40, 0.80) | 0.60 (0.40, 0.80) | 0.919 |
| monocyte absolute value | 0.37 (0.30, 0.45) | 0.39 (0.32, 0.48) | 0.001 |
| large platelet ratio | 29.20 (24.50, 34.20) | 28.90 (24.50, 34.20) | 0.791 |
| mean corpuscular hemoglobin | 30.10 (28.90, 31.10) | 30.20 (29.00, 31.00) | 0.548 |
| red blood cell volume distribution width standard deviation | 40.50 (38.80, 42.20) | 40.75 (38.90, 42.50) | 0.178 |
| hemoglobin | 139.00 (130.00, 153.00) | 145.00 (133.00, 157.00) | ＜0.001 |
| corpuscular specific volume | 41.80 (39.30, 45.20) | 43.25 (39.90, 46.60) | ＜0.001 |
| mean corpuscular volume | 89.50 (86.70, 92.00) | 89.60 (86.50, 91.80) | 0.707 |
| mean corpuscular hemoglobin concentration | 334.00 (327.00, 341.00) | 335.50 (330.00, 342.00) | 0.017 |
| coefficient of variation of red blood cell distribution width | 12.40 (12.00, 13.10) | 12.50 (12.10, 13.20) | 0.086 |
| blood platelet count | 251.00 (216.00, 287.00) | 248.00 (213.00, 283.75) | 0.334 |
| mean platelet volume | 10.50 (9.90, 11.10) | 10.53 (9.62, 11.44) | 0.629 |
| platelet distribution width | 12.70 (11.50, 14.50) | 12.90 (11.40, 14.93) | 0.454 |
| absolute basophil count | 0.03 (0.02, 0.05) | 0.03 (0.02, 0.05) | 0.163 |
| absolute eosinophil count | 0.10 (0.06, 0.17) | 0.10 (0.06, 0.19) | 0.445 |
| neutrophil percentage | 55.92 (48.10, 63.75) | 56.62 (48.61, 64.63) | 0.096 |
| lymphocyte percentage | 34.40 (29.50, 39.20) | 33.83 (26.35, 41.31) | 0.083 |
| red blood cell count | 4.70 (4.39, 5.14) | 4.88 (4.47, 5.29) | 0.001 |
| white blood cell count | 5.73 (4.90, 6.65) | 6.03 (5.28, 7.02) | ＜0.001 |
| uric acid | 295.90 (251.30, 359.30) | 324.95 (270.73, 383.48) | ＜0.001 |
| burea nitrogen | 4.10 (3.52, 4.82) | 4.25 (3.63, 5.03) | 0.015 |
| creatinine | 68.70 (60.30, 83.00) | 74.00 (62.90, 85.75) | ＜0.001 |
| thyrotropin | 2.04 (1.47, 2.80) | 2.15 (1.59, 2.84) | 0.167 |
| transparency |  |  | 0.457 |
| clear | 267 (86.13%) | 1828 (84.28%) |  |
| slightly turbid | 26 (8.39%) | 238 (10.97%) |  |
| turbid | 10 (3.23%) | 57 (2.63%) |  |
| severe turbidity | 0 (0%) | 3 (0.14%) |  |
| urinary bilirubin |  |  | 0.413 |
| negative | 300 (96.77%) | 2113 (97.42%) |  |
| weak positive | 0 (0%) | 3 (0.14%) |  |
| positive | 3 (0.97%) | 10 (0.46%) |  |
| urine ketone body |  |  | 0.145 |
| negative | 288 (92.90%) | 1992 (91.84%) |  |
| weak positive | 1 (0.32%) | 40 (1.84%) |  |
| positive | 14 (4.52%) | 94 (4.33%) |  |
| urinary leukocyte esterase |  |  | 0.035 |
| negative | 269 (86.77%) | 1763 (81.28%) |  |
| weak positive | 15 (4.84%) | 168 (7.75%) |  |
| positive | 19 (6.13%) | 195 (8.99%) |  |
| urinary occult blood |  |  | 0.414 |
| negative | 226 (72.90%) | 1519 (70.03%) |  |
| weak positive | 63 (20.32%) | 475 (21.9%) |  |
| positive | 14 (4.52%) | 132 (6.09%) |  |
| color |  |  | 0.695 |
| light amber | 4 (1.29%) | 16 (0.74%) |  |
| light yellow | 77 (24.84%) | 611 (28.17%) |  |
| red | 1 (0.32%) | 2 (0.09%) |  |
| tan | 1 (0.32%) | 7 (0.32%) |  |
| yellow | 152 (49.03%) | 994 (45.83%) |  |
| wheat yellow | 37 (11.94%) | 300 (13.83%) |  |
| dark amber | 0 (0%) | 3 (0.14%) |  |
| dark yellow | 12 (3.87%) | 68 (3.14%) |  |
| colorless | 19 (6.13%) | 125 (5.76%) |  |
| specific gravity | 1.02 (1.01, 1.02) | 1.02 (1.01, 1.02) | 0.089 |
| pH | 6.00 (5.50, 6.50) | 6.00 (5.50, 6.50) | 0.058 |
| urine nitrite |  |  | 1.000 |
| negative | 302 (97.42%) | 2117 (97.60%) |  |
| positive | 1 (0.32%) | 9 (0.41%) |  |
| urinary protein |  |  | 0.971 |
| negative | 278 (89.68%) | 1950 (89.90%) |  |
| weak positive | 14 (4.52%) | 94 (4.33%) |  |
| positive | 11 (3.55%) | 82 (3.78%) |  |
| urine glucose |  |  | 0.651 |
| negative | 303 (97.74%) | 2120 (97.74%) |  |
| weak positive | 0 (0%) | 3 (0.14%) |  |
| positive | 0 (0%) | 3 (0.14%) |  |
| urobilinogen |  |  | 0.419 |
| negative | 286 (92.26%) | 1976 (91.10%) |  |
| positive | 17 (5.48%) | 150 (6.92%) |  |
| alpha-fetoprotein | 2.53 (1.79, 3.45) | 2.64 (2.00, 3.67) | 0.024 |
| glucose | 4.85 (4.61, 5.12) | 4.88 (4.61, 5.13) | 0.554 |

P < 0.050 is considered statistical significance. Categorical variables, expressed as frequencies (proportions), line χ2 test. Non-normally distributed variables, expressed as median (interquartile range), line Mann–Whitney U test.

**Supplementary Table 2** Predictive performance of different base learners and different number of features

| Model | Number of Features | Accuracy | AUC | Sensitivity | Specificity |
| --- | --- | --- | --- | --- | --- |
| LR | 2 | 0.63 | 0.64 | 0.51 | 0.64 |
|  | 4 | 0.66 | 0.74 | 0.72 | 0.65 |
|  | 6 | 0.68 | 0.77 | 0.74 | 0.68 |
|  | 8 | 0.69 | 0.78 | 0.75 | 0.68 |
|  | 10 | 0.71 | 0.80 | 0.76 | 0.70 |
|  | 12 | 0.71 | 0.80 | 0.77 | 0.70 |
|  | 14 | 0.71 | 0.80 | 0.77 | 0.70 |
|  | 16 | 0.71 | 0.80 | 0.76 | 0.71 |
|  | 18 | 0.71 | 0.80 | 0.73 | 0.70 |
|  | 20 | 0.71 | 0.79 | 0.73 | 0.70 |
| SVM | 2 | 0.51 | 0.62 | 0.59 | 0.60 |
|  | 4 | 0.58 | 0.61 | 0.59 | 0.58 |
|  | 6 | 0.59 | 0.62 | 0.59 | 0.60 |
|  | 8 | 0.61 | 0.64 | 0.60 | 0.61 |
|  | 10 | 0.62 | 0.68 | 0.63 | 0.62 |
|  | 12 | 0.61 | 0.68 | 0.64 | 0.61 |
|  | 14 | 0.64 | 0.70 | 0.66 | 0.64 |
|  | 16 | 0.65 | 0.71 | 0.63 | 0.65 |
|  | 18 | 0.65 | 0.71 | 0.66 | 0.64 |
|  | 20 | 0.64 | 0.70 | 0.67 | 0.64 |
| XGBoost | 2 | 0.63 | 0.75 | 0.75 | 0.62 |
|  | 4 | 0.69 | 0.81 | 0.82 | 0.67 |
|  | 6 | 0.68 | 0.82 | 0.82 | 0.66 |
|  | 8 | 0.68 | 0.82 | 0.82 | 0.67 |
|  | 10 | 0.70 | 0.84 | 0.81 | 0.68 |
|  | 12 | 0.70 | 0.84 | 0.84 | 0.68 |
|  | 14 | 0.70 | 0.84 | 0.84 | 0.68 |
|  | 16 | 0.70 | 0.84 | 0.84 | 0.68 |
|  | 18 | 0.70 | 0.84 | 0.84 | 0.68 |
|  | 20 | 0.70 | 0.84 | 0.84 | 0.68 |
| RF | 2 | 0.70 | 0.75 | 0.62 | 0.72 |
|  | 4 | 0.74 | 0.82 | 0.72 | 0.75 |
|  | 6 | 0.73 | 0.82 | 0.73 | 0.73 |
|  | 8 | 0.72 | 0.84 | 0.82 | 0.71 |
|  | 10 | 0.71 | 0.83 | 0.77 | 0.70 |
|  | 12 | 0.72 | 0.83 | 0.77 | 0.71 |
|  | 14 | 0.71 | 0.82 | 0.79 | 0.70 |
|  | 16 | 0.71 | 0.82 | 0.75 | 0.70 |
|  | 18 | 0.71 | 0.82 | 0.78 | 0.71 |
|  | 20 | 0.71 | 0.81 | 0.79 | 0.69 |
| KNN | 2 | 0.60 | 0.73 | 0.73 | 0.59 |
|  | 4 | 0.67 | 0.74 | 0.70 | 0.66 |
|  | 6 | 0.69 | 0.75 | 0.71 | 0.69 |
|  | 8 | 0.70 | 0.76 | 0.70 | 0.70 |
|  | 10 | 0.71 | 0.78 | 0.74 | 0.70 |
|  | 12 | 0.71 | 0.79 | 0.74 | 0.70 |
|  | 14 | 0.70 | 0.79 | 0.78 | 0.69 |
|  | 16 | 0.71 | 0.78 | 0.76 | 0.69 |
|  | 18 | 0.70 | 0.78 | 0.75 | 0.70 |
|  | 20 | 0.71 | 0.78 | 0.74 | 0.70 |

**Supplementary Table 3** Prediction performance of different machine learning models with different sample ratio

| **Participant number** | **Models** | **Accuracy (mean±SD)** | **AUC (mean±SD)** | **Sensitivity (mean±SD)** | **Specificity (mean±SD)** |
| --- | --- | --- | --- | --- | --- |
| dyslipidemia: 310,  non-dyslipidemia: 1550 | LR | 0.71±0.01 | 0.79±0.02 | 0.77±0.04 | 0.69±0.02 |
|  | RF | 0.74±0.01 | 0.83±0.03 | 0.80±0.05 | 0.73±0.01 |
|  | KNN | 0.71±0.01 | 0.79±0.02 | 0.77±0.04 | 0.70±0.01 |
|  | SVM | 0.62±0.12 | 0.72±0.06 | 0.72±0.07 | 0.59±0.16 |
|  | XGBoost | 0.73±0.02 | 0.85±0.01 | 0.83±0.04 | 0.70±0.03 |
|  | Ensemble Model | 0.78±0.02 | 0.87±0.01 | 0.77±0.04 | 0.78±0.03 |
| dyslipidemia: 310, non-dyslipidemia: 775 | LR | 0.72±0.04 | 0.81±0.03 | 0.76±0.04 | 0.71±0.06 |
|  | RF | 0.75±0.05 | 0.84±0.03 | 0.79±0.05 | 0.73±0.06 |
|  | KNN | 0.74±0.04 | 0.82±0.03 | 0.78±0.05 | 0.72±0.05 |
|  | SVM | 0.72±0.03 | 0.80±0.03 | 0.75±0.05 | 0.70±0.06 |
|  | XGBoost | 0.75±0.03 | 0.84±0.03 | 0.81±0.05 | 0.73±0.06 |
|  | Ensemble Model | 0.78±0.03 | 0.87±0.02 | 0.78±0.04 | 0.79±0.05 |
| dyslipidemia: 310, non-dyslipidemia: 516 | LR | 0.74±0.02 | 0.80±0.02 | 0.77±0.05 | 0.72±0.06 |
|  | RF | 0.75±0.03 | 0.84±0.02 | 0.80±0.02 | 0.73±0.05 |
|  | KNN | 0.74±0.03 | 0.81±0.02 | 0.76±0.05 | 0.72±0.07 |
|  | SVM | 0.73±0.02 | 0.80±0.02 | 0.77±0.04 | 0.71±0.06 |
|  | XGBoost | 0.75±0.04 | 0.84±0.02 | 0.77±0.04 | 0.73±0.07 |
|  | Ensemble Model | 0.77±0.03 | 0.85±0.03 | 0.77±0.03 | 0.77±0.05 |
| dyslipidemia: 310, non-dyslipidemia: 387 | LR | 0.73±0.02 | 0.81±0.02 | 0.77±0.07 | 0.70±0.05 |
|  | RF | 0.75±0.04 | 0.83±0.03 | 0.80±0.07 | 0.70±0.04 |
|  | KNN | 0.74±0.03 | 0.81±0.03 | 0.78±0.04 | 0.71±0.04 |
|  | SVM | 0.73±0.02 | 0.81±0.03 | 0.77±0.07 | 0.70±0.06 |
|  | XGBoost | 0.75±0.03 | 0.84±0.03 | 0.78±0.05 | 0.73±0.04 |
|  | Ensemble Model | 0.76±0.03 | 0.85±0.02 | 0.76±0.05 | 0.77±0.05 |
| dyslipidemia: 310, non-dyslipidemia: 310 | LR | 0.72±0.05 | 0.80±0.04 | 0.75±0.10 | 0.69±0.07 |
|  | RF | 0.74±0.02 | 0.82±0.02 | 0.76±0.04 | 0.72±0.06 |
|  | KNN | 0.74±0.04 | 0.81±0.03 | 0.77±0.07 | 0.70±0.05 |
|  | SVM | 0.72±0.04 | 0.80±0.03 | 0.75±0.09 | 0.68±0.08 |
|  | XGBoost | 0.75±0.04 | 0.83±0.02 | 0.81±0.06 | 0.69±0.07 |
|  | Ensemble Model | 0.74±0.04 | 0.82±0.02 | 0.76±0.05 | 0.71±0.04 |
